# Voluntary Hospitals and Poor Law Infirmaries

**Published:** 1924-02

**Authors:** E. W. Morris

**Affiliations:** C.B.E.


### VOLUNTARY HOSPITALS AND POOR LAW INFIRMARIES

### ADDRESS BY MR. E. W. MORRIS, C.B.E.

On January 16 Mr. E. W. Morris, C.B.E., House-Governor of the London Hospital, addressed the Guild of Pharmacists on "The Future of the Voluntary and Municipal Hospitals." Considerations of space admit only of our giving such passages as bear immediately upon the important question of the relief of hospital pressure by utilising vacant beds in the Poor-Law Infirmaries. Mr. Morris began by pointing out that, although the Waiting List at the London now stands at 865, at the Whitechapel Infirmary opposite there are scores of empty beds.

### Efficiency of the Infirmaries.

The buildings (Mr. Morris continued) were so near that interchange of staff seemed an easy undertaking. We knew that some of the infirmaries were becoming so splendidly staffed and so efficiently equipped that they were treating with success the same type of case as was entering the voluntary hospitals; they had operating theatres and facilities for sterilisation equal to those of the most up-to-date hospital; some of them had their X-ray, bacteriological and clinical-pathological departments; their patients were separated into groups convenient for treatment, medical and surgical, chronic and acute; some of them were securing the services of the same consultants and specialists as were visiting the voluntary institutions. Indeed their efficiency, in some cases, was so obvious that I was once told by a very important Government official that the time was approaching when the voluntary hospitals would have to state clearly what was the reason for their continuance.

### Infirmaries Two-thirds Empty.

According to the Cave Committee there are 117 voluntary hospitals in London, and in the rest of Great Britain there are 835, making a total of 952. These hospitals have, in London, 12,797 beds, and in the rest of Great Britain 39,397, making a total of 52,194 beds. What about the infirmaries? They are changing their names for some reason or other —and that is worth noting. They come under the Destitution Authority, and it is this taint of the Poor-law which makes them unpopular with the poor, and they are reminded of this taint in that, except in emergency, they must enter the infirmary *via* the relieving officer, and when admitted must wear the infirmary garb. Every hospital man knows the difficulty of removing a patient from a hospital to a Poor-law infirmary—the patient hates it. Also every hospital man has got into trouble at some time or other by sending a patient direct to the infirmary and omitting to tell him that he must go to the relieving officer first. There are about 92,000 beds in the Poor-law infirmaries of Great Britain, and we have been told that about 30,000 of these are empty in the summer and 20,000 in the winter. It seemed reasonable to try and devise some means by which these unused beds could be utilised to ease the everincreasing pressure on the voluntary hospitals by persuading the sick poor to make use of the infirmary as well as the hospital; a most difficult undertaking under present conditions, for many of the sick prefer to go untreated than to become paupers.

### Stepney's Refusal.

We considered a scheme at the "London" whereby for a time Whitechapel Infirmary should take the emergencies of the district so that we could be free to get on with the waiting list; we were to take a group of eighty beds and were actually going to pay for cases admitted to these beds and sent on from the hospital. Our consultants were quite ready to go over and operate or advise if and when desired. The scheme did not materialise for reasons that need not be gone into now, but it is worthy of note that the most powerful opposition came from the Public Health Committee of Stepney. . . . At the rate of two and a-half beds per 1,000 of population (various authorities differ as to the number required, from 1·5 to 5) there should be about 160,000 hospital beds, so that there is a lack of about 108,000. Part of this deficiency could be made up by using the empty beds in the Poor-law infirmaries.

### Degrees of Destitution.

In 1910 a circular letter was issued by the Local Government Board to the Guardians to the following effect:—"In determining this question (whether a person is destitute or not) the Guardians have to remember that a person may be destitute in respect of the want of some particular necessity of life without being destitute in all respects, as, for instance, a person who is not destitute in the sense that he is entirely devoid of the means of subsistence, may yet be destitute in that he is unable to provide for himself the particular form of medical attendance or treatment of which he is in urgent need." From this it follows that if a hospital has filled its beds and cannot admit any more cases—say accidents and other emergencies, such emergencies, if they needed institutional treatment beyond their means, would have to be admitted to the Poor-law infirmary. Such cases, however, being "destitute" so far as Medical treatment was concerned, would have to pass through the hands of the relieving officer (unless immediately urgent) and would wear the infirmary dress on admission.

### Avoiding the Stigma.

It is now permitted by the Ministry of Health that persons who are not destitute may enter a Poor-law institution for treatment on paying the cost of treatment—maintenance and overhead charges. Such a case is not admitted by the relieving officer, but by the medical officer of the infirmary, or the clerk to the Guardians (in some cases by the matron) and on the recommendation in some cases of a general practitioner. The Ministry permits—in fact advocates—the calling in of consultants. I have before me short reports from various medical officers in various Poor-law infirmaries from all parts of the kingdom on the working of this arrangement which show that under certain circumstances a patient may go into a Poor-law infirmary and be entirely free of all stigma of Poor-law, although actually treated in an institution which is under the Destitution Authority. The voluntary Hospitals could ease the pressure on their beds by declining to admit accidents until they had caught up their waiting lists and forcing these emergencies to be taken elsewhere—the infirmary. The police and the county council would be instructed to take accidents to the nearest infirmary.

### Special Terms for Hospital Cases.

But if cases may be admitted on special terms—free of all Poor-law taint—as they now can be (by payment of cost) and secure thereby certain amenities, would it not be possible to arrange for cases sent from the hospital to secure the same convenience by other "special terms"? The hospitals are now taking hundreds of cases which would be in the infirmaries but for the hospitals. Could not Poor-law reciprocate and allow that cases sent by the hospital, who have passed the hospital inquiry officers or almoners, should be grouped together in the infirmary, should escape the relieving officer, should wear their own clothes, should have more freedom for visiting, and be registered in special books? The hospital could not pay in cash, but it could pay in other ways. The consultants would probably visit, and would be glad to do so if they could take their students; the scientific laboratories, the X-ray departments could be put at the service of the infirmary. Qualified hospital students would be glad to act as house surgeons and house physicians under the infirmary assistant medical officers. There could be free interchange of patients.

### Why Not Experiment?

We must remember that the infirmary has to take the sick poor and the existence of the hospital near by does not free it of its duties. But a friendly arrangement such as I have suggested would be infinitely better than to stand on our rights. The growth of the Hospital Saving Association makes some such arrangement more necessary than ever. It is not right that a sick patient belonging to this Association should be turned away from the hospital simply because there is no bed. The hospitals have a right to use selective powers, and if they cannot take all cases they must choose those who have been thrifty enough to try and prepare for the day when they should need the hospital's help. I am assuming that we all recognise that medical urgency is of course the first consideration. If the Ministry of Health would not agree to payment "by service" as I have suggested, then the balance could be struck at the end of the year out of the Hospital Saving Association grant, which will amount to three guineas a week for every patient we admit of that Association. I should like the Ministry of Health's permission to try the experiment for one year, and I am certain the Guardians and we could draw up rules which would be of benefit to the patients (especially those poor folk waiting for the letter that is so long in coming telling them there is at last a bed), to the infirmary and to the hospital.

